# Pediatrics

**Published:** 1896-09

**Authors:** Maud Josephine Frye

**Affiliations:** Clinical instructor in diseases of children, Medical Department, University of Buffalo


### Progress in Medical Science.

### PEDIATRICS.

Reported by MAUD JOSEPHINE FRYE, M. D.,
Clinical instructor in diseases of children, Medical Department, University of Buffalo.

### ANTITOXIN IN PRIVATE PRACTICE.

AS A SUPPLEMENT to the number of July 1, 1896, *Pediatrics* publishes the report of committee appointed by the American Pediatric Society to investigate this subject. The committee, consisting of Drs. L. Emmett Holt, W. P. Northrup, Joseph O'Dwyer and Samuel S. Adams, submitted to the society, at its eighth annual meeting at Montreal in May, a report, of which the following is a summary :

1. The report includes returns from 615 physicians. Of this number more than 600 have pronounced themselves as strongly in favor of the serum treatment, the great majority being enthusiastic in its advocacy.

2. The cases included have been drawn from localities widely separated from each other, so that any peculiarity of local conditions, to which might be ascribed the favorable reports, must be excluded.

3. The report includes the record of every case returned, except those in which the evidence of diphtheria was clearly questionable. It will be noted that doubtful cases which recovered have been excluded, while doubtful cases which were fatal have been included.

4. No new cases of sudden death immediately after injection have been returned.

5. The number of cases injected reasonably early, in which the serum appeared not to influence the progress of the disease, was but nineteen, these being made up of nine cases of somewhat doubtful diagnosis ; four cases of diphtheria complicating measles, and three malignant cases in which the progress was so rapid that the cases had passed beyond any reasonable prospect of recovery before the serum was used. In two of these the serum was of uncertain strength and of doubtful value.

6. The number of cases in which the patients appeared to have been made worse by serum were three, and among these there is only one new case in which the result may fairly be attributed to the injection.

7. The general mortality in the 5,794 cases reported was 12.3 per cent.; excluding the cases moribund at the time of injection or dying within twenty-four hours, it was 8.8 per cent.

8. The most striking improvement was seen in the cases injected during the first three days. Of 4,120 such cases the mortality was 7.3 per cent.; excluding cases moribund at the time of injection or dying within twenty-four hours, it was 4.8 per cent.

9. The mortality of 1,448 cases injected on or after the fourth day was 27 per cent.

10. The most convincing argument, and to the minds of the committee an absolutely unanswerable one, in favor of serum therapy is found in the results obtained in the 1,256 laryngeal cases (membranous croup). In one-half of these recovery took place without operation, in a large proportion of which the symptoms of stenosis were severe. Of the 533 cases in which intubation was performed the mortality was 25.9 per cent., or less than half as great as has ever been reported by any other method of treatment.

11. The proportion of cases of broncho-pneumonia—5.9 per cent.—is very small and in striking contrast to results published from hospital sources.

12. As against the two or three instances in which the serum is believed to have acted unfavorably upon the heart, might be cited a large number in which there was a distinct improvement in the heart's action after the serum was injected.

13. There is very little, if any, evidence to show that nephritis was caused in any case by the injection of serum. The number of cases of genuine nephritis is remarkably small, the deaths from that source numbering but fifteen.

14. The effect of the serum on the nervous system is less marked than upon any other part of the body; paralytic sequelæ being recorded in 9.7 per cent. of the cases, the reports going to show that the protection afforded by the serum is not great unless injections are made very early.

### ACTION OF THE SOCIETY UPON THE REPORT.

At the close of its presentation, the society voted to accept the report of the committee, and after a full discussion it was decided to embody its conclusions in the following resolutions:

1. *Dosage.*—For a child over two years old, the dosage of antitoxin should be in all laryngeal cases with stenosis, and in all other severe cases, 1,500 to 2,000 units for the first injection, to be repeated in from eighteen to twenty-four hours if there is no improvement; a third dose after a similar interval, if necessary. For severe cases in children under two years old, and for mild cases over that age, the initial dose should be 1,000 units, to be repeated as above if necessary; a second dose is not usually required. The dosage should always be estimated in antitoxin units, and not of the amount of serum.

2. *Quality of Antitoxin.*—The most concentrated strength of an absolutely reliable preparation.

3. *Time of Administration.*—Antitoxin should be administered as early as possible on a clinical diagnosis, not waiting for a bacteriological culture. However late the first observation is made, an injection should be given, unless the progress of the case is favorable and satisfactory.

The committee was appointed to continue its work for another year, and was requested to issue another circular asking for the further coöperation of the profession, this circular to be sent out as soon as possible, in order that physicians may record their cases as they occur through the coming year.

---

### PASTEURISATION OF MILK.

FREEMAN (*Archives of Pediatrics*, August, 1896,) has devised an apparatus whereby the Pasteurisation of milk can be done at a temperature ranging from 65° to 70° C. The same steriliser can be used for rapid cooling, an important feature of low temperature sterilisation and one often overlooked. The low temperature used destroys almost all the ordinary air bacteria which occur commonly in milk, and also the bacillus tuberculosis, the bacillus typhosis, the bacillus diphtheria, and many other pathogenic bacteria. It causes no change in the taste of the milk and avoids those chemical changes which are produced by higher temperatures. The milk should only be used during the twenty-four hours following the Pasteurisation.

